# Approach to a Child with Hypophosphatemia

**DOI:** 10.3390/biom15091321

**Published:** 2025-09-15

**Authors:** Agnieszka Antonowicz, Patryk Lipiński, Michał Popow, Piotr Skrzypczyk

**Affiliations:** 1 Department of Pediatrics and Nephrology, Medical University of Warsaw, 02-091 Warsaw, Poland; agnieszka.antonowicz-zawislak@wum.edu.pl; 2 Institute of Clinical Sciences, Maria Skłodowska-Curie Medical Academy, 00-136 Warsaw, Poland; patryk.lipinski.92@gmail.com; 3 Department of Internal Medicine and Endocrinology, Medical University of Warsaw, 02-091 Warsaw, Poland; michal.popow@uckwum.pl

**Keywords:** hypophosphatemia, children, phosphate, rickets, tubulopathy, X-linked hypophosphatemia

## Abstract

Hypophosphatemia is a rare ion disorder in children, but it carries the risk of serious clinical sequelae in tissues and organs with high energy requirements, such as bone tissue. This article discusses the metabolism of phosphate in the body, the clinical manifestations of hypophosphatemia, and the diagnostic tests necessary in patients with this disorder. Extra-renal causes are analyzed, and renal forms of hypophosphatemia are discussed in detail. Renal hypophosphatemia, depending on the mechanism, is divided into PTH-dependent (e.g., primary hyperparathyroidism), FGF23-dependent (e.g., X-linked hypophosphatemia), and intrinsic renal hypophosphatemia (e.g., Fanconi syndrome). The treatment of hypophosphatemia involves compensating for phosphate deficiency, often simultaneously with the supply of an active form of vitamin D. Always seek causal treatment, such as parathyroidectomy in primary hyperparathyroidism. In the FGF-23-dependent forms of X-linked hypophosphatemia and tumor-induced osteomalacia, burosumab has proven to be an effective and safe drug. **Conclusions:** a child with hypophosphatemia requires a multidisciplinary approach and determination of the mechanism of phosphate deficiency in the body.

## 1. Phosphates and Their Role in the Body’s Homeostasis

### 1.1. Phosphate Demand and Turnover in the Human Body

Phosphates constitute approximately 0.5% of the body weight of newborns and approximately 1% of the body weight of older children. About 85% of phosphates in the human body are found in bones and teeth, mainly in the form of hydroxyapatite, 14% is intracellular fluid, and only about 1% of phosphates are found in extracellular fluid (including serum) [1]. Only 30% of the 1% of circulating phosphorus mentioned is detected by laboratory tests. This is inorganic phosphorus, meaning it is not bound to a carbon atom [2].

**Figure 1:**
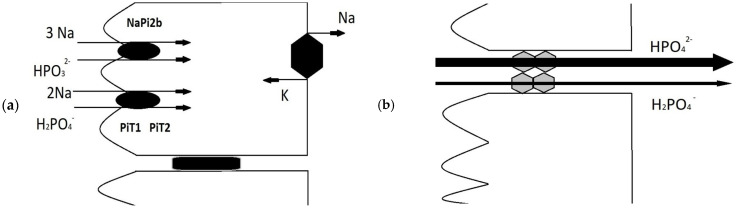
Intestinal transport of phosphate; (**a**) transcellular transport carried by NaPi2b, PIT-1, and PIT-2 transporters; (**b**) paracellular transport driven by claudins.

The daily dietary intake of phosphates is approximately 1200 mg. Intestinal absorption is approximately 800 mg/day, and intestinal secretion of phosphates is 150 mg/day. Bone resorption and bone formation in the phosphate range are the same and amount to 300 mg/day. The glomerular filtration of phosphates is 6000 mg/day. The tubular reabsorption of phosphates is 5350 mg/day, while only 650 mg/day is excreted in urine; 550 mg of phosphates are removed daily with feces [3]. The diets of modern Europeans and Americans, unlike those of previous centuries, are rich in inorganic phosphate, and the amount of phosphate needed to maintain homeostasis is readily supplied through the diet. Excess phosphate in the diet is particularly hazardous for individuals with impaired kidney function, as it leads to the systemic accumulation of phosphate. Inorganic phosphorus is absorbed from the gastrointestinal tract by two pathways: paracellular and transcellular (Figure 1). Paracellular transport is carried out by claudin group proteins, is driven by a chemical gradient, and is non-saturable. Transcellular transport is an active transport regulated by the active form of vitamin D (calcitriol) and is saturable [2,4].

In people with efficient kidneys, excess dietary phosphate is excreted through the mechanisms described below. Table 1 presents examples of dietary products rich in phosphates [3,5,6,7].

### 1.2. Role of Phosphate in the Human Body

Phosphates, although as mentioned, make up only 1% of the human body’s content, are essential for virtually all life processes. In the first place, energy processes—phosphates are a source of readily available energy accumulated in adenosine triphosphate (ATP) and participate in the construction of nucleic acids.

The roles of phosphates in the body are the construction of cell membranes (phospholipids) and nucleic acids (DNA, RNA) and hydroxyapatite—the mineral component of bones and teeth, activation of many enzymes and, together with calcium, the coagulation system, participation in intracellular energy metabolism (adenosine triphosphate, ATP), participation in intracellular signaling—“second transmitter” (cAMP, phosphatidylinositol), buffering of intracellular fluids (maintenance of proper pH–isohydria), participation in oxygen transport to tissues—release of oxygen from hemoglobin (2,3-diphosphoglycerate, 2,3-DPG) and involvement in immune processes [3,5,6,7].

Phosphates and calcium are the main mineral components of bone tissue. The mineralization process involves the formation of hydroxyapatite from phosphates and calcium around collagen fibers. The process of mineralization is strictly regulated by many cells (osteoblasts, osteoclasts) and proteins (e.g., osteoprotegerin, RANKL, bone morphogenetic proteins, osteopontin) [8,9].

The growth plate of long bones is composed of successive layers of chondrocytes, which proliferate, undergo hypertrophy, and then undergo apoptosis. Mineralization and bone tissue formation occur in the layer of hypertrophic chondrocytes. Phosphates are essential for the process of chondrocyte apoptosis; therefore, their deficiency leads to the inhibition of chondrocyte apoptosis, growth plate widening, impaired bone matrix mineralization, and the development of rickets [10,11].

## 2. Mechanisms of Phosphate Homeostasis

PTH, 1,25(OH)_2_D, and FGF23 are involved in the regulation of phosphate metabolism.

### 2.1. Parathyroid Hormone

Parathyroid hormone (PTH) is the primary hormone regulating calcium and phosphate metabolism. It is produced in the parathyroid glands. It maintains homeostasis by releasing calcium and phosphate from bones, reducing calcium excretion and phosphate reabsorption in the kidneys, and indirectly increasing calcium and phosphate absorption in the intestines. PTH inhibits phosphate reabsorption in the proximal tubule by decreasing the expression of sodium-phosphate cotransporters NaPi2a and NaPi2c. PTH, by increasing the production of calcitriol, stimulates indirectly the expression of the NaPi2b in the intestine and thus enhances the absorption of phosphate from food [4]. Another action of PTH that affects calcium and phosphate metabolism is the stimulation of the production of active vitamin D by increasing the activity of CYP27B1, leading to increased hydroxylation of 25(OH)D to the most active form—1,25(OH)_2_D [12].

The stimulation of bone resorption is indirect since osteoclasts do not have a receptor for PTH. The hormone binds to osteoblasts, increasing their expression of RANKL and inhibiting their secretion of osteoprotegerin (OPG). Free OPG competitively binds to RANKL as a decoy receptor, preventing RANKL from interacting with RANK, a receptor for RANKL. The binding of RANKL to RANK stimulates osteoclast precursors and bone resorption. PTH, through a cAMP-dependent mechanism, inhibits the activity of NHE3 (sodium hydrogen exchanger 3) in the proximal tubule, reducing the loss of hydrogen ions, which play a crucial role in the RANKL metabolic pathway [13].

### 2.2. Calcitriol–1,25(OH)_2_D

Sources of cholecalciferol are animal foods (rich sources are, for example, fish like eels) and dermal synthesis under UV radiation, and sources of ergocalciferol are plant foods. Once synthesized or absorbed in the intestine, vitamin D2 and D3 undergo the same metabolic processes. The first step involves hydroxylation at position 25 in the liver during the so-called first pass. The resulting 25(OH)D is the main store of vitamin D in the body, and commonly available tests determine its concentration, which indicates the body’s vitamin D reserves. The hydroxylation step at position 25 is not regulated and depends primarily on substrate supply.

The 25(OH)D then undergoes a second hydroxylation at the 1-alpha position, resulting in the most metabolically active form of vitamin D–1,25(OH)_2_D–calcitriol, considered by many to be a hormone rather than a vitamin. 1-alpha-hydroxylase is encoded by the *CYP27B1* gene and is primarily expressed in the kidneys. Its activity is tightly regulated, with PTH increasing and FGF23 decreasing its activity. Vitamin D is mainly inactivated by the attachment of another -OH group at position 24 and then excreted in the urine. Additional unregulated sources of calcitriol can be granulomas in diseases such as sarcoidosis, brucellosis, or untreated tuberculosis. Macrophages/monocytes that form granulomas exhibit high 1-alpha-hydroxylase activity, which can lead to excessive vitamin D in these patients. The calcium and phosphorus effects of calcitriol are mediated by its binding to a vitamin D receptor (VDR) [14].

The main action of calcitriol is to increase blood calcium and phosphate levels by promoting the absorption of dietary calcium and phosphate from the gastrointestinal tract. Calcitriol increases NaPi2b protein expression and sodium-dependent phosphate intestinal uptake. It also stimulates the release of calcium and phosphate from bone, acting on osteoblasts and increasing their production of RANKL, which in turn activates osteoclasts. Finally, calcitriol increases renal tubular calcium reabsorption, not significantly affecting phosphate reabsorption [4].

The fact that calcitriol increases bone resorption seems unintuitive and counterintuitive to the observed clinical effects. However, it appears that in a situation of adequate calcium supply (intestinal absorption), the production of new bone tissue outweighs bone resorption. Hence, the in vivo effect of calcitriol under the condition of dietary calcium supply is to increase bone mass.

### 2.3. Fibroblast Growth Factor 23

Fibroblast growth factor 23 (FGF23) is a 251-amino-acid protein, discovered in 2000 by a team of Japanese researchers, which is most similar to FGF19 and FGF21 [15]. Fibroblast growth factor 23 (FGF23) is considered by many to be the most essential player in phosphate metabolism. FGF23 is mainly produced in osteocytes and osteoblasts, although initially its presence was described in the brain and thymus [15]. It is regarded as the most potent phosphaturic factor or hormone (i.e., a biological agent that increases urinary phosphate wasting). Within a year of its discovery, it was found that excess FGF23 is responsible for the occurrence of many diseases with renal phosphate wasting, including autosomal dominant hypophosphatemic rickets (ADHR), in which pathogenic variants of the *FGF23* gene have been identified [16]. There are numerous regulators of FGF23 release, among which serum phosphate concentration is the most essential one (Table 2).

FGF23 is responsible for regulating calcium and phosphate metabolism by binding to the FGF23 receptors (FGF23Rs)—FGFR1c, FGFR3c, and FGFR4. To activate FGFR1c, a cofactor (Klotho protein) is necessary. FGF23 reduces intestinal phosphate absorption by inhibiting 1,25(OH)_2_D alpha-hydroxylase (action mediated by binding to FGFR3c and FGFR4 in proximal tubule). Also, FGF23 increases phosphate excretion in urine by inhibiting the expression of NaPi2a and NaPi2c on the apical membrane of proximal tubular cells (action mediated by binding to FGFR1c with Klotho in proximal tubule). In addition, it lowers serum 1,25(OH)_2_D levels by stimulating 24-hydroxylase activity [19,20].

The *kl* gene and klotho protein were first described in mice [21]. Klotho protein deficiency leads to premature aging and increased organ calcification, including in the arteries. It is believed that low KLOTHO protein concentration, combined with very high FGF23 concentrations and hyperphosphatemia, is partly responsible for adverse changes in the circulatory system in patients with chronic kidney disease [22,23].

A summary of the direct actions of key players on phosphate metabolism is presented in Table 3.

## 3. Symptoms of Hypophosphatemia

Moderate hypophosphatemia is usually asymptomatic. Symptoms are typically observed when serum phosphate levels fall below 1 mg/dL (i.e., <0.32 mmol/L). The tissues and organs most sensitive to hypophosphatemia are those that are energetically active, such as muscles, the liver, the central nervous system, the peripheral nervous system, and blood cells. Chronic hypophosphatemia may manifest itself as muscle weakness, bone and joint pain, paresthesia, muscle tremors, convulsions, proximal myopathy, myalgia, vitamin D-resistant rickets, short stature, osteomalacia, liver dysfunction, heart rhythm disorders, and hemolysis [3,5,7,24]. Regarding bone symptoms, in children, chronic hypophosphatemia leads to hypophosphatemic rickets, and in adults, it causes osteomalacia (Table 4).

## 4. Diagnostic Tests in the Diagnosis of Hypophosphatemia

### 4.1. Overview of Laboratory Tests

Table 5 shows the tests performed during the diagnosis of hypophosphatemia. In addition to standard laboratory blood tests determining calcium and phosphate metabolism (ions, ALP, PTH, 25(OH)D and 1,25(OH)_2_D concentrations, kidney function parameters) and urine (urine analysis, crystallization indices), we also calculate indices such as glomerular filtration rate (GFR), fractional urinary phosphate excretion (TRP), total phosphate reabsorption (TmP/GFR) [3,7,25]. A key element in correctly interpreting the results is to relate them to reliable reference values. In 2024, phosphate metabolism standards developed in a group of 455 children (from birth to adulthood) based on the LMS method were published (the HARP study). The authors established standards for seven key elements of phosphate metabolism: serum phosphate, urinary phosphate-to-creatinine ratio, urinary calcium-to-creatinine ratio, TRP, TmP/GFR, serum intact FGF23, and serum soluble Klotho [26].

### 4.2. Assessment of Serum Phosphate Concentration

Phosphate concentration is highest in newborns, then gradually decreases, with a further significant decrease occurring during puberty—faster in girls, which corresponds to their earlier onset of puberty. It is essential to refer phosphate concentrations to the appropriate standards. Many standards for serum phosphate concentrations have been published, including two large-scale studies in recent years: the HARP [26] and the CALIPER studies [27]. Normal serum phosphate concentrations in children, as reported by various authors, are presented in Table 6.

### 4.3. Assessment of Urinary Phosphate Excretion

Regarding the evaluation of the urine test, the recommendations emphasize the usefulness of indicators calculated from urine portions rather than daily urine collections, as daily urinary phosphate excretion is more a derivative of diet than of the mechanisms controlling its homeostasis. As for the urine portion, traditionally it was considered that the authoritative portion is the second portion after the night. Although the current view is that any portion can be assessed, it is worth noting that the authors used the second portion to establish standards [26].

To assess phosphate excretion in urine, we calculate the fractional excretion of phosphate (FePO_4_), tubular reabsorption of phosphate (TRP), and the TMP/GFR ratio.

To calculate the fractional excretion of phosphate in urine, we use the following formula:

FePO_4_ = 100 * (U_P_ * Crea)/(P * U_Crea_)

where serum phosphate—P, urinary phosphate—U_P_, serum creatinine—Crea, urinary creatinine—U_Crea_.

To calculate phosphate reabsorption, we use the following formula:

TRP = 1 − FePO_4_

TmP/GFR is the tubular maximum reabsorption of phosphate related to glomerular filtration rate.

To calculate all of the above indicators, you can use the calculator available on the website of the European Society for Pediatric Nephrology (https://www.espn-online.org/tmp-gfr-calculator/#calculator (accessed on 12 September 2025)). The authors used as reference values the already quoted standards of Pott et al. [26].

### 4.4. Assessment of FG23 Concentration

Many commercially available tests measure FGF23 concentration (immunoassays). C-terminal assays measure both the concentration of the entire molecule and the inactive C-terminal fragment (cFGF23); intact assays measure only the concentration of the whole, active molecule [29]. The latter (intactFGF23-iFGF23) is recommended for diagnostic purposes [29], especially since different clinical conditions can impact the serum cFGF23/iFGF23 ratio. As mentioned, reliable iFGF23 standards have been developed for children. The researchers used a commercially available enzyme-linked immunosorbent assay (ELISA) kit from Quidel [26]. When interpreting intact FGF23 concentrations, it is essential to consider factors that may affect interpretation, as patients with chronic kidney disease typically have repeatedly elevated FGF23 concentrations from the early stages onwards. Other factors to consider include, e.g., iron deficiency and inflammation in the body (Table 2) [17,18]. Interpretation of FGF23 concentrations can be challenging, especially in patients with FGF23-independent renal hypophosphatemia who have experienced a decrease in glomerular filtration (e.g., patients with Fanconi syndrome and chronic kidney disease in the course of cystinosis).

### 4.5. Molecular Studies

Genetic (molecular) testing is recommended in most cases of renal hypophosphatemia and plays a vital role in the diagnosis of calcium-phosphate metabolism disorders, including hypophosphatemia. This is particularly important because clinical analysis, pedigree analysis, biochemical parameters, and imaging tests may not always determine the cause of the disease, especially in cases of a mild phenotype (e.g., heterozygotes of pathogenic variants in SCL34A1 and SCL34A3) and negative family history. As disease phenotypes may overlap, for example, in the cases of autosomal dominant hypophosphatemic rickets (ADHR) and X-linked hypophosphatemia (XLH), genetic panels using next-generation sequencing to evaluate multiple genes are recommended [30]. In the absence of results, it is advisable to search for microdeletions using microarray technology. In addition, as targeted therapy has become available for XLH, a correct diagnosis is necessary before treatment initiation [25].

### 4.6. Imaging Studies

In terms of diagnostic imaging, a standard ultrasound is an essential test. The examination allows assessment of the size and structure of the kidneys. It is necessary to evaluate the kidneys for the presence of calcifications in both the parenchyma and the urinary tract. Calcifications indicate that hyperphosphaturia is also accompanied by hypercalciuria [25].

X-rays of the wrist and knee joints are necessary for features indicative of rickets on physical examination or elevated alkaline phosphatase activity. The images taken should be evaluated using the Rickets Severity Score scale [31].

## 5. Causes of Hypophosphatemia

The causes of hypophosphatemia are shown in Table 7 and in Supplementary Table S1. Depending on the evaluation of renal phosphate wasting, the causes of hypophosphatemia can be divided into non-renal and renal causes. Decreased TmP/GFR indicates renal phosphate wasting. Conversely, hypophosphatemia with fractional excretion of phosphate below 5.0% is an indicator of extra-renal phosphate loss [25]. Among the renal causes, we distinguish between PTH and FGF23-dependent and PTH and FGF23-independent causes.

## 6. Non-Renal Causes of Hypophosphatemia

### 6.1. Hypophosphatemia Secondary to Insufficient Supply or Impaired Absorption of Phosphates

This phenomenon is relatively rare, as already mentioned; diets are usually abundant in phosphate. Hypophosphatemia in this mechanism may occur: in newborns with low birth weight who are exclusively breastfed, children fed with elemental or hypoallergenic formulas, during inadequate parenteral nutrition, when using high doses of phosphate binders (calcium carbonate, calcium acetate, sevelamer), after gastrointestinal surgery (blind loops, short bowel syndrome, bariatric surgery), due to infective agents (intestinal tuberculosis, giardiasis, Whipple’s disease), mucosal abnormalities (celiac disease), inflammatory bowel disease (Crohn’s syndrome) or infiltrative conditions (amyloidosis, lymphoma) [3,7,24,25,32]. Laboratory tests reveal hypophosphatemia, hypophosphaturia, and high alkaline phosphatase levels (Table 8).

### 6.2. Loss of Phosphates During Renal Replacement Therapy

Hyperphosphatemia is one of the primary symptoms of chronic kidney disease. It may also occur in patients with acute kidney injury. Dialysis modalities vary depending on their potential for phosphate removal, and the elimination of phosphate by dialysis is a cornerstone of managing hyperphosphatemia. In peritoneal dialysis, phosphate mass removal appears to be correlated with peritoneal creatinine but not urea clearance. Several factors, including blood and dialysate flow rates, dialyzer membrane surface area, and ultrafiltration volume, influence phosphate clearance during hemodialysis. Phosphate removal can be substantially improved by hemodiafiltration, increased dialysis frequencies, and extended treatment times [35].

Continuous renal replacement therapies (CRRT), especially those using convectional transport (hemofiltration, hemodiafiltration), are very effective in phosphate removal. Hypophosphatemia during CRRT usually occurs after 48 h; therefore, it is necessary to supplement it in the diet or parenteral nutrition. The appropriate selection of fluids during continuous renal replacement therapy (CRRT) is also essential. Traditional fluid used in the Baxter/Gambro system (Hemosol) contains no phosphate. In contrast, the replacement fluid used in the citrate anticoagulation system (Phoxilium) is enriched in phosphate (1.2 mmol/L), which leads to lower serum phosphate levels [36,37,38].

### 6.3. Hypophosphatemia Due to Intracellular Shift

The shift of phosphate from the blood (into cells, bone) rarely leads to clinically significant hypophosphatemia. Various mechanisms lead to this condition. After insulin infusion, intracellular transport of glucose, potassium, and phosphate occurs. Phosphates are consumed very rapidly in the refeeding syndrome [3,7].

Hungry bone syndrome is a condition characterized by profound hypocalcemia and hypophosphatemia that may persist for a long time. This condition most commonly occurs following parathyroidectomy (due to primary or secondary hyperparathyroidism). Hungry bone syndrome occurs due to a sudden shift in bone metabolism after the removal of excess parathyroid hormone, resulting in increased osteoblastic activity and rapid consumption of calcium and phosphorus by the bones. Several factors, including patient age and the duration and severity of preexisting hyperparathyroidism, can affect its presentation [39,40].

## 7. Renal Causes of Hypophosphatemia

### 7.1. PTH-Dependent Renal Causes

#### 7.1.1. Primary Hyperparathyroidism

Primary hyperparathyroidism is rare in pediatrics. It is not infrequently associated with a genetic background. In patients, uncontrolled parathyroid hormone production leads to increased bone resorption and hypercalcemia. Parathormone is a potent phosphaturic factor; therefore, patients exhibit hypophosphatemia, hyperphosphaturia, and decreased tubular maximum for phosphate per glomerular filtration rate (TmP/GFR). Hypercalciuria is secondary to hypercalcemia and bone resorption (Table 9). Patients exhibit elevated 1,25(OH)_2_D concentrations due to the activating effect of PTH. Due to hypercalciuria with hyperphosphaturia, patients are at risk for nephrolithiasis and nephrocalcinosis. Examples of genetically caused hyperparathyroidism in children include MEN (multiple endocrine neoplasia) types 1–5, hyperparathyroidism-jaw tumor, neonatal severe primary hyperparathyroidism, and familial isolated hyperparathyroidism [41,42].

#### 7.1.2. Calcipenic Rickets

The typical calcipenic rickets is vitamin D deficiency rickets. It is extremely rare in Europe today. It can be, for example, a consequence of a misdiagnosed hypersensitivity to vitamin D (based on a rapidly overgrowing fontanel) and withdrawal of vitamin D supplementation. Children with vitamin D deficiency may develop rickets. It manifests as a syndrome of changes characterized by reduced bone mineralization, abnormal bone development, and progressive skeletal deformities, including bowlegs, knock knees, rib widening (also known as a “rose chest”), chest deformity, skull bulging, and kyphoscoliosis. Vitamin D deficiency leads to impaired intestinal calcium absorption, resulting in secondary hyperparathyroidism and, consequently, urinary phosphate wasting (Table 9) [3,12,18,25].

In vitamin-D-dependent rickets, a defect in vitamin D metabolism leads to an impaired vitamin D function despite a normal vitamin supply, resulting in clinical symptoms resembling those of deficiency rickets (Table 10 and Table 11) [25,32,43,44].

### 7.2. PTH- and FGF23-Independent Renal Causes

In this group of diseases characterized by renal phosphate loss, there is a primary disruption of proximal tubule function in phosphate reabsorption. This is either an isolated genetic defect or a generalized defect involving differentially expressed functions of all proximal tubules. The concentration of PTH, especially FGF23, is usually decreased secondary to hypophosphatemia (Table 12) [3,7,24,25,44].

#### 7.2.1. Hypophosphatemia Due to SLC34A1 and SLC34A3 Pathogenic Variants

*SLC34A1* and *SLC34A3* genes encode sodium-phosphate cotransporters in the proximal tubule (NaPi2a and NaPi2c, respectively). The clinical and biochemical spectrum of diseases associated with defects in these genes is heterogeneous (Table 13). In general, increased phosphate loss leads to the inhibition of FGF23 secretion, resulting in unblocked 1-alpha-hydroxylase activity, inadequate elevation of 1,25(OH)_2_D levels, and increased gastrointestinal calcium absorption. Secondary to the positive calcium balance, some patients develop hypercalciuria [3,7,24,25].

Not in all patients with biallelic pathogenic variants in *SLC34A1* and *SLC34A3* can hypophosphatemia be observed. Patients could develop hyperphosphaturia, hypercalciuria, nephrolithiasis, nephrocalcinosis, and osteoporosis.

Biallelic pathogenic *SLC34A1* is related to infantile hypercalcemia-2 (HCINF2; #616963). In these patients, hypercalcemia is observed in the neonatal and infantile periods, which resolves with age [45]. However, patients have an increased risk of kidney stones, nephrocalcinosis, and chronic kidney disease (CKD) [46].

Hereditary hypophosphatemic rickets with hypercalciuria (HHRH; #241530) is an autosomal recessive disorder caused by biallelic pathogenic variants of the *SLC34A3* gene, and characterized by phosphaturia, low TMP/GFR, and high 1,25(OH)_2_D concentrations. Serum phosphate concentrations may be reduced or normal. It can be distinguished from other forms of hypophosphatemia by increased serum levels of 1,25(OH)_2_D, resulting in hypercalciuria. In some patients, the first symptom of the disease could be the presence of urinary tract stones [47,48,49].

A multi-center European analysis of the clinical course in patients with pathogenic variants in these genes was published this year. The authors analyzed 113 patients with pathogenic or likely pathogenic variants in *SLC34A1* or *SLC34A3*. Patients affected with biallelic SLC34A1 variants showed polyuria, failure to thrive, vomiting, constipation, hypercalcemia, and nephrocalcinosis in infancy. In contrast, patients affected with biallelic *SLC34A3* variants presented in childhood or even adulthood with rickets/osteomalacia and/or osteopenia/osteoporosis, hypophosphatemia, and, less frequently, nephrocalcinosis, while the prevalence of kidney stones was comparable. Adult patients with an *SLC34A3* defect had a six-fold increase in CKD prevalence compared to the general population. Both groups of patients, with *SLC34A1* or *SLC34A3* defects, shared a common biochemical pattern, including elevated 1,25(OH)_2_D and alkaline phosphatase levels, suppressed parathyroid hormone (PTH), and hypercalciuria. Heterozygous carriers showed similar but less pronounced phenotypes [30].

The following case from our clinical practice illustrates the complexity of the phenotype of patients with *SLC34A1* and *SLC34A3* variants. A 13-year-old girl was referred due to recurrent nephrolithiasis (two episodes of renal colic, numerous deposits up to 4 mm in the right calyx-pelvis system). Diagnostic tests revealed normocalcemia (2.4 mmol/L), phosphate concentration in the lower range of normal (1.1 mmol/L), low 25OHD concentration—21.3 ng/mL, and elevated 1,25(OH)_2_D—103 pg/mL, mild hypercalciuria (4.5 mg/kg/24 h i.e., 0.113 mmol/kg/24 h), 24 h urinary calcium-creatinine ratio—0.26 mg/mg, i.e., 0.735 mol/mol), and decreased TmP/GFR—0.82 mmol/L (normal range: 0.88–1.52). Genetic testing was performed—gene panel using next-generation sequencing, revealing a potentially pathogenic variant in one allele of the *SLC34A3* gene (c.1093 + 1G > C). Considering the mechanism of hypercalciuria, treatment with a low-dose phosphate mixture (0.4 mL/kg/24 h in divided doses) was initiated. After 9 days, the girl developed an allergic reaction in the form of a generalized rash, which completely resolved after discontinuation of treatment. No attempt was made to restart treatment, and the girl remains on a low-sodium, normal-calcium, and high-phosphate diet. No new deposits/calcifications are forming in the urinary tract, and calcium urinary excretion remains normal (3.5 mg/kg/24 h, i.e., 0.088 mmol/kg/24 h).

#### 7.2.2. Fanconi Syndrome

Fanconi syndrome is a congenital or acquired complex dysfunction of the proximal tubule of the nephron, characterized by impaired reabsorption of amino acids, glucose, phosphate, bicarbonate, uric acid, and other substances, resulting in their massive loss in the urine and associated clinical sequelae. Fanconi syndrome is a polyetiological disorder. Inborn, genetically determined metabolic diseases, as well as acquired conditions, drugs, or toxins, can lead to its occurrence. Fanconi syndrome extremely rarely develops as an idiopathic form [50,51,52,53,54] (Table 14).

Patients present with variously expressed growth retardation, polyuria, polydipsia, a tendency to dehydration, vomiting, anorexia, constipation, muscle weakness, and bone disorders: hypophosphatemic rickets in children and osteomalacia in adults. In addition, there are symptoms typical of the underlying condition.

Laboratory tests reveal glucosuria, mild proteinuria in urinalysis, hypophosphatemia, hyperchloremia, hypokalemia, hyponatremia, metabolic acidosis, and a tendency to hypouricemia (Table 15). In some cases (e.g., cystinosis), progression towards end-stage kidney disease is observed. Apart from hypophosphatemia, patients with calcium-phosphorus disorders may present with low 1,25(OH)_2_D (resulting from disturbed hydroxylation due to tubular dysfunction). In cases of renal function impairment, elevated parathyroid hormone levels are also observed [50,51,52,53,54].

### 7.3. FGF23-Dependent Renal Causes

In this group of disorders, there is impaired physiological regulation and overproduction of FGF23 and, as a result, decreased hydroxylation of vitamin D at the 1-alpha position and decreased phosphate absorption in the gastrointestinal tract, as well as decreased expression of NaPi2a and NaPi2c cotransporters in the proximal tubule, resulting in loss of phosphate in the urine. FGF23 levels are in “inadequate”, normal, or elevated levels. The conditions can be acquired or congenital. The most common of these diseases is X-linked hypophosphatemia (XLH) (Table 16).

Elevated intact FGF23 concentrations in children with hypophosphatemia might seem to indicate FGF23-dependent renal phosphate wasting, e.g., autosomal dominant hypophosphatemic rickets, X-linked hypophosphatemia, or tumor-induced osteomalacia [15,55,56]. In a recent study evaluating a cohort of pediatric and adult patients with different causes of renal hypophosphatemia, a cut-off point for intact FGF23 of 27 pg/mL was 100% sensitive and specific in distinguishing FGF23-dependent from FGF23-independent hypophosphatemia [29].

#### 7.3.1. Tumor-Induced Osteomalacia

Tumor-induced osteomalacia (TIO) is a paraneoplastic syndrome secondary to a mesenchymal tumor, occurring mainly in adults. Tumors are usually benign, small, and mesenchymal in origin and are primarily located in soft tissues or bones. Tumors can be located anywhere in the body, from the head to the toes, making localization challenging. In 60% of cases, the presence of the *FN1/FGFR1* or *FN1/FGF1* fusion gene (TIO molecular marker) is found. Tumors produce FGF23 or other phosphaturic factors. The most common symptoms include bone pain, fractures, and muscle weakness [57,58,59].

Biochemical analysis reveals a pattern of disturbances similar to XLH with dominating hypophosphatemia, hyperphosphaturia, low concentration of 1,25(OH)_2_D, and elevated or “inadequately” normal concentration of FGF23. Functional imaging using somatostatin receptor-based PET imaging (68Ga DOTA-based technologies) is the first-line investigation, which should be followed by CT or MRI-based anatomical imaging. Once localized, complete surgical excision is the treatment of choice, which brings dramatic resolution of symptoms [57,58,59].

As an example, we present an adult patient from our clinical practice whose diagnosis began with frequent fractures. A 48-year-old woman with a history of a single wrist fracture in childhood following a fall while skating and multiple low-energy fractures of both cortical and trabecular bone over the past six years was referred due to severe symptomatic hypophosphatemia (muscle weakness), severe pain in her long bones that reduced her quality of life, and multiple low-energy fractures. The bone fragility developed seven years ago without any apparent cause. The most serious fractures concerned: C6 and C7 spinous processes during a sneeze seven years ago, femoral neck fracture in 2 years, linear fracture of both iliac plates (without obvious trauma), bilateral rib fractures (6, 9 and 10) during a cough three years ago, spontaneous fracture of the manubrium of the sternum in two years ago and of the body of the sternum before a year. Laboratory tests revealed hypophosphatemia up to 0.46 mmol/L with a significantly reduced TmP/GFR (0.320 mmol/L, normal range: 0.88–1.42), and a reduced mean phosphate reabsorption capacity of 69.6% (normal range: 85–95%). Despite hypophosphatemia, a high FGF23 level of 133 kRU/l persisted (normal value: 26–110). The overall clinical picture was consistent with acquired TIO. The source of FGF23 secretion was identified using PET scanning with Gallium-labeled somatostatin analogs. It was a mesenchymal tumor of the right mandibular ramus. After its resection, phosphatemia returned to normal, and bone pain significantly decreased. Due to the high risk of incomplete tumor removal, the patient remains under constant endocrinological supervision.

#### 7.3.2. Cutaneous Skeletal Hypophosphatemia Syndrome

Cutaneous skeletal hypophosphatemia syndrome (CSHS) is an ultra-rare somatic mosaic RASopathy in which somatic RAS-activating variants in regions of skeletal dysplasia produce excessive FGF23 [58,60,61]. Bone fractures, limb deformities, and rickets/osteomalacia represent the consequences of a disrupted phosphorus homeostasis (FGF23-induced hypophosphatemia) and RAS-mediated malformations of bone development. CSHS is also included in a group of epidermal nevus syndromes characterized by a hallmark skin lesion (epidermal nevi) in association with developmental abnormalities of other organ systems [58,60,61].

#### 7.3.3. Iron-Induced Hypophosphatemia

The interaction between iron supply, gene expression, FGF23 secretion, and calcium-phosphate metabolism is complex. Iron deficiency causes an increase in FGF23 gene expression. On the other hand, iron carboxymaltose (FCM) has been shown to impair FGF23 removal and increase phosphaturia. This effect was not observed with the supply of other preparations; moreover, with long-term supply, it is offset by the inhibition of FGF23 gene expression [62,63].

In vivo, the effect of iron inhibition of gene expression seems to prevail. A 2021 meta-analysis showed that iron supply reduced FGF23 levels, and this effect was more pronounced with oral supply [64].

#### 7.3.4. X-Linked Hypophosphatemia

X-linked hypophosphatemia (XLH; OMIM #307800) is a rare genetic disease occurring in 1 in 20,000 to 25,000 births. It is a genetically determined condition, caused by pathogenic variants in the *PHEX* gene, characterized by a chronic disease of the skeletal system marked by excessive phosphate loss through the kidneys due to increased (or inadequate) expression and activity of FGF23 [43]. This disease is the most common form of genetically determined hypophosphatemic rickets. It is also one of the rare human diseases that is inherited in an X-linked dominant manner.

*PHEX* pathogenic (loss-of-function) variant(s) result in the overexpression of FGF23, leading to the abnormalities described above. Additionally, an excess of proteins involved in bone metabolism, such as SIBLING proteins (e.g., osteoprotegerin), is implicated in the pathogenesis of the disease [10]. In healthy people, these proteins are inactivated by the active phosphate-regulating neutral endopeptidase (PHEX) protein. In addition, some of the symptoms (e.g., craniosynostosis) result from an excess of FGF23 and its binding to receptors in connective tissue [33].

The symptoms of XLH are heterogeneous and change over time due to the progressive nature of the disease. In children, delayed and disproportionate growth, craniosynostosis, rickets, delayed motor development, and gait disorders are observed. Both children and adults may experience bone and joint pain, short stature, and limb deformities (especially varus deformity of the lower limbs). Periapical abscesses, excessive tooth decay, osteomalacia, joint stiffness, muscle pain and weakness, Chiari malformations, and gait disorders. Patients experience a severely reduced quality of life. Unlike adults, hearing loss is not commonly observed in children. In adults, we also see more fractures (including stress fractures and pseudofractures), osteoarthritis, extraosseous calcifications (including enthesopathies, spinal stenosis), and progressive disability affecting the ability to work [65]. A tremendous burden is the muscle weakness resulting from chronic hypophosphatemia [33,66].

The disease is suspected based on typical biochemical abnormalities (Table 17). In children, a diagnosis of XLH should be considered in the presence of clinical, biochemical, or radiological signs of rickets, impaired growth velocity, and hypophosphatemia associated with isolated renal phosphate wasting and in the absence of vitamin D or calcium deficiency, especially in the case of a positive family history [47]. In patients with a negative family history (20–30% of patients), genetic testing is necessary to differentiate from other forms of hypophosphatemic rickets. A targeted panel of genes sequenced by next-generation sequencing is recommended first. However, it should be noted that some patients may have specific molecular genetic defects, such as large deletions, deletions that remove pseudo-exons of PHEX, or mosaicism [33].

We present the case of a girl with XLH who underwent two corrective orthopedic procedures before her calcium and phosphate metabolism was examined, and she was referred to our center for diagnosis. A 3-year-old girl was found to have lower limb varus deformity since the age of one and a half. The patient has been under orthopedic care, initially receiving conservative treatment with increased doses of vitamin D (up to 2000 IU/day). Due to severe deformation of the lower limbs, at the age of 2 years and 8 months, the Eight-Plates Guided Growth Systems (Orthofix, Lewisville, TX, USA) were placed on the proximal ends of both tibial epiphyses. At the age of 3 years, the Eight-Plate on the left side was revised. It was only after the second orthopedic surgery that the girl’s calcium and phosphate levels were tested, revealing normocalcemia (calcium—2.32 mmol/L), hypophosphatemia (phosphorus—0.85 mmol/L), high vitamin D concentration (25OHD—85.4 pg/mL), high alkaline phosphatase activity (460 IU/L) and decreased TmP/GFR—0.99 mmol/L (normal values: 1.32–1.85). Genetic testing using next-generation sequencing revealed a pathogenic variant in one allele of the *PHEX* gene (c.1601C > T). No pathogenic variants were found in the other genes tested (*CLCN5*, *DMP1*, *ENPP1*, *FAH*, *FGF23*, *KL*, *SLC34A1*, *SLC34A3*, *VDR*). The family history was unremarkable, and no genetic testing was performed on the parents. The girl began treatment with alfacalcidol (33.3 ng/kg/24 h) and a phosphate mixture (1.1 mL/kg/24 h). The treatment was very poorly tolerated (vomiting, abdominal pain), and bone pain and deformities persisted. Due to the unavailability of burosumab in our country at that time, individual consent for treatment was requested from the National Health Fund. Treatment was started at the age of 4 years and 9 months, initially at a dose of 10 mg s.c. every 2 weeks (0.5 g/kg), then, due to a decrease in phosphate concentration, the dose was increased to 20 mg s.c. In the meantime, it became possible to treat a larger number of patients with this drug in our country (the Ministry of Health drug program). The patient has now been treated for 3 years, and bone deformities have regressed. Her growth is between the 10th and 25th percentile; she has normophosphatemia, normal TmP/GFR values, and no treatment complications have been observed.

#### 7.3.5. Autoantibodies Against PHEX

In January 2025, a fascinating study was published, analyzing 13 adults with FGF23-dependent hypophosphatemia. Other causes of renal phosphate loss (including genetic causes and TIO) were ruled out in these patients. Five of the 13 patients were found to have autoantibodies against the PHEX protein. The molecular effect resembles XLH—the lack of active PHEX protein leads to the overexpression of the FGF23 gene, resulting in hyperphosphaturia and hypophosphatemia. One of the patients was also found to have other autoimmune diseases, including autoimmune thyroiditis, idiopathic thrombocytopenic purpura, systemic lupus erythematosus, and antiphospholipid syndrome [67]. Although this is only one report, and all patients were adults from Japan, autoantibodies against PHEX may explain cases of renal hypophosphatemia when other causes are excluded.

## 8. Treatment of Hypophosphatemia

### 8.1. Causative Treatment

In all cases of hypophosphatemia, causal treatment—aimed at addressing the cause of phosphate deficiency—should be pursued. Hence, making a diagnosis is crucial. Complete elimination of the cause is possible in only a few situations (e.g., removal of a tumor in TIO or removal of the cause of malabsorption syndrome). In other cases, symptomatic treatment remains the preferred approach. Examples of causal treatment of hypophosphatemia are shown in Table 18.

### 8.2. Symptomatic Treatment

Most patients with hypophosphatemia can only be treated symptomatically. The mainstay is the supplementation of lost phosphate. The drug of choice is a phosphate mixture. The typical composition of the mix is the following (in Latin):

*Natrium phosphoricum monobasicum 4.5 g;*

*Natrium phosphoricum dibasicum 36.25 g;*

*Aqua dest. ad 250 g*

*m.f. solutio.*

The mixture is dosed per kg of body weight (20–60 (80) mg/kg/24 h or 0.7–2.0 mmol/kg/24 h or 0.4–1.25 mL/kg/24 h). The drug requires multiple doses a day. In many situations of massive phosphate loss (e.g., XLH), it is not possible to normalize the serum phosphate concentration, so other indicators, such as a decrease in alkaline phosphatase activity, should be used to assess the effectiveness of treatment [30].

To reduce the risk of PTH increase, patients require an active form of vitamin D (alfacalcidol—1-alpha-OH-vitamin D or calcitriol—1,25(OH)2-vitamin D). Alfacalcidol is usually administered at a dose of 30–50 ng/kg/24 h [30]. Note that administration of active forms of vitamin D increases calcium absorption, and thus, secondarily, calciuria. Hypercalciuria, together with pre-existing hyperphosphaturia, increases the risk of nephrocalcinosis.

Patients with hypophosphatemic rickets require a multidisciplinary approach to treatment. Often, orthopedic treatment is necessary, although surgical treatment is recommended once a reduction in the severity of rickets lesions has been achieved. Patients with XLH may require neurosurgical (Chiari malformation), ENT (osteosclerosis), and dental (caries, periapical abscesses) treatment. Multispecialty surveillance is necessary [30].

Patients with Fanconi syndrome require a supply of water, sodium, potassium, and sometimes calcium, in addition to phosphate supplementation and an active form of vitamin D.

### 8.3. Burosumab

Burosumab is a human recombinant monoclonal antibody directed against FGF23. It is produced using a cell line derived from Chinese hamster ovary cells. By inhibiting FGF23, burosumab increases tubular reabsorption of phosphate in the kidneys and serum 1,25(OH)_2_D concentrations.

Greater safety and security of the drug with conventional therapy specified in adult patients with XLH (with additional drug once every 4 weeks) [68]. Then, a study in children showed more stable phosphate concentrations with a supply once every 2 weeks compared to a supply once every 4 weeks [69]. A subsequent study demonstrated significantly higher efficacy of the drug administered once every 2 weeks compared to conventional treatment, as assessed by improvements in bone health, as indicated by the RSS scale and the 6-min walking test (6MWT) [70].

Adverse events were mild (mainly related to the injection site) and did not require treatment discontinuation in any patient. Antibodies to burosumab developed in some patients, but their presence did not affect the effectiveness of the treatment [70].

Burosumab has also been successfully used in adults with TIO, where the drug normalized phosphatemia, reduced bone symptoms, and significantly improved quality of life [58,71].

In 2018, burosumab was approved by the European Medicines Agency in Europe and the U.S. Food and Drug Administration for the treatment of children and adults with XLH and TIO.

In the following years, additional studies on burosumab in XLH were published, indicating the long-term effects of treatment [52]. These studies also demonstrated the beneficial effects of the drug on other aspects of XLH, such as the prevention of dental abscesses [72]. In August 2024, a comprehensive meta-analysis and systematic review of the available clinical trials on burosumab treatment for pediatric XLH showed its significant therapeutic effectiveness, particularly in improving key biochemical markers and achieving significant skeletal improvements, as indicated by RSS and 6MWT [53].

Given the common underlying role of FGF23-mediated hypophosphatemia in XLH, TIO, and CSHS, burosumab also became a promising therapeutic option for patients with CSHS. To date, several reports have been published regarding both adults and children with CHCS treated with burosumab [39,54,55].

In the authors’ country, the drug has been available under the so-called drug program for children with XLH since 2024. Individual consent for reimbursement of treatment in other registered indications can be applied for.

### 8.4. Limitations of Available Treatment Methods and New Therapies on the Horizon

Unfortunately, all available treatments for hypophosphatemia have their limitations and can cause various complications, some of which are serious.

Treatment with a phosphate mixture is associated with several complications. First, the drug is poorly tolerated—many patients experience troublesome gastrointestinal symptoms (abdominal pain, nausea, diarrhea). In addition, the supply of phosphate is a potent stimulus of PTH, which can cause secondary hyperparathyroidism [30] observed in up to 30% of patients receiving conventional treatment [73,74,75].

The administration of active forms of vitamin D increases calcium absorption, and thus, secondarily, calcemia and calciuria. Hypercalciuria, together with pre-existing hyperphosphaturia, increases the risk of nephrocalcinosis and urolithiasis [73,74,75].

Burosumab has undoubtedly become a breakthrough in the treatment of FGF23-dependent hypophosphatemia. Like any therapy, it is not without side effects. In recent years, three systematic reviews with meta-analyses have been published discussing the efficacy and safety of burosumab in both children and adults. The conclusions from these analyses are as follows: the incidence of adverse effects in patients receiving burosumab is higher compared to those receiving placebo; however, the effects are mild (most commonly injection site reaction events, arthralgia, and headaches) and self-limiting, and do not lead to discontinuation of therapy. The studies did not observe a clinically significant increase in calcemia, calciuria, PTH concentration, or the severity of nephrocalcinosis [73,74,75].

However, the authors emphasize the short observation period. Considering the mechanism of action of burosumab, it is necessary to conduct studies that closely monitor calcium and phosphate metabolism in treated patients and the occurrence of extraosseous calcifications, including those in the kidneys, arteries, and heart. In addition, it is not clear whether burosumab can normalize patients’ height and how it affects already-present extraosseous calcifications, enthesopathies, dental abscesses, and hearing impairment. It is worth noting that there is also a lack of data on efficacy and safety in the youngest children—those under 1 year of age. Furthermore, it should be remembered that burosumab will likely need to be administered for life, which raises questions about cost-benefit analysis [73,74,75]. In the authors’ country, only children are eligible for treatment with burosumab. Adults are currently treated under individual consent from the National Health Fund. In many countries, the drug is still not available.

Many children with XLH continue to have growth deficiency despite treatment with burosumab. Recombinant human growth hormone may be considered for these patients [30], although the literature shows no apparent beneficial effect of this treatment on the final height of children with XLH [76,77]. Patients with XLH exhibit hyperactivity of ERK and MAPK signaling pathways. In a mouse model, blocking the MAPK pathway improved growth and rickets and, notably, almost normalized the growth plate organization [78]. There are no human trials yet, although MAPK inhibitors are already used in the treatment of cancers, including melanoma [79].

Gene therapy is another promising therapeutic approach to monogenic forms of hypophosphatemia. A liver-targeting adeno-associated virus (AAV) inhibiting FGF23 signaling has recently been developed. An AAV vector carrying a gene for the C-terminal fragment of FGF23 was injected into mice with XLH. The hepatic expression of the C-terminal fragment of FGF23 corrected skeletal manifestations and osteomalacia in these animals [80]. In potential human treatment, it appears that gene therapy should be administered as early as possible to prevent osseous and extraosseous complications [81].

In a study by Imel et al., iron supplementation in three adult patients with ADHR and iron deficiency resulted in normalization of serum phosphorus levels, alleviation of symptoms, and allowed patients to discontinue calcitriol and phosphate supplementation [82]. Further studies (including multicenter studies) involving larger groups of patients with this diagnosis are undoubtedly necessary. There is no doubt that iron metabolism should be monitored in these patients. Assessing the body’s iron supply and supplementation in case of deficiency appears to be beneficial in any condition of FGF23 excess, including chronic kidney disease [64].

## 9. Conclusions

Hypophosphatemia is a decrease in phosphate concentration below the normal values. The symptoms of hypophosphatemia affect mainly tissues and organs with high energy demand (muscles and nervous system) and bone tissue. In children, chronic hypophosphatemia leads to growth retardation and hypophosphatemic, vitamin-D-resistant rickets. In older children and adults, chronic hypophosphatemia causes osteomalacia, fractures, and pseudofractures.

Laboratory diagnostics must include a complete assessment of calcium-phosphorus metabolism with calculation of the following indices: FePO_4_, TRP, and TmP/GFR. Depending on whether increased urinary phosphate loss is detected, hypophosphatemia can have either renal or extrarenal causes. Renal causes can be divided into intrinsic renal causes (e.g., Fanconi syndrome), parathyroid hormone-dependent causes (e.g., calcipenic rickets), and fibroblast growth factor 23-dependent causes (e.g., X-linked hypophosphatemia).

Treatment involves both symptomatic measures (primarily phosphate supplementation) and causative measures (i.e., treatment of the underlying disease, such as cysteamine in patients with cystinosis). Burosumab is a monoclonal antibody targeting FGF-23 approved for the treatment of X-linked hypophosphatemia and tumor-induced osteomalacia. Clinical experience gathered in recent years indicates the extraordinary effectiveness and safety of the drug compared to conventional therapy.

## Figures and Tables

**Table 1:** Phosphate-rich food.

Product	Phosphorus Content Per 100 g
Wheat bran	950 mg
Sesame	720 mg
Parmesan	694 mg
Gouda cheese	546 mg
Cheddar cheese	545 mg
Egg yolk of a hen	542 mg
Soybeans (grain)	603 mg
Turkey	200 mg
Red caviar	490 mg
Black caviar	465 mg
Shrimp	225 mg
Horseradish (root)	130 mg
Garlic	100 mg
Oxalis (green)	90 mg

**Table 2:** Factors influencing fibroblast growth factor-23 production according to [17,18].

Positive Regulator	Negative Regulator
Increased serum phosphate	Low serum phosphate
Increased 1,25(OH)_2_D (calcitriol)	Low serum calcium
Parathyroid hormone (PTH) increases FGF23 secretion; however, it activates the cleavage of the intact molecule into C-terminal and N-terminal FGF23 peptides.	Low 1,25(OH)_2_D (calcitriol)
Deficiency of PHEX	Loss-of-function variants in *GALNT3*
Deficiency of DMP1	
Deficiency of FAM20C	
O-glycosylation by GALNT3	
Iron deficiency Erythropoetin Inflammation Hypoxia	

**Table 3:** The summary of the direct action of parathyroid hormone, calcitriol, and fibroblast growth factor-23 on phosphate metabolism.

Site of Action	Parathyroid Hormone	Calcitriol	Fibroblast Growth Factor 23
Intestine	-	Increases intestinal phosphate absorption by increasing expression of sodium-phosphate cotransporter (NaPi2b)	-
Kidney	Increases urinary phosphate wasting by decreasing expression of sodium-phosphate cotransporters (NaPi2a and NaPi2c)	-	Increases urinary phosphate wasting by decreasing expression of sodium-phosphate cotransporters (NaPi2a and NaPi2c)
Bone	Increases bone resorption by stimulating osteoblasts to produce RANKL, which, in turn, binds to RANK and stimulates osteoclasts	Increases bone resorption	-

**Table 4:** Symptoms of hypophosphatemia.

Muscles	Nervous System	Skeletal System	Other Systems
Proximal myopathy Muscle weakness Myalgia Impaired respiratory muscle function Rhabdomyolysis	Paresthesia Muscle tremors Mental disorders Convulsions Coma	Youngest children: vitamin D-resistant rickets and short stature Older children and adults: pain and bone complications—osteomalacia and pathological fractures	Liver dysfunction Cardiac contractility and rhythm disorders Hemolysis Leukocyte dysfunction Thrombocyte dysfunction

**Table 5:** Diagnostic tests performed in hypophosphatemia according to [25], modified.

Blood	Total calcium, magnesium, phosphates Ionized calcium urea, creatinine, uric acid Acid-base balance Albumin alkaline phosphatase alanine and aspartate transaminases (ALT, AST) Bone fraction of alkaline phosphatase (when elevated ALT and AST) intact parathyroid hormone metabolites of vitamin D: 1,25(OH)2D, 25OHD intact fibroblast growth factor 23
Urine	Urine analysis potassium, sodium, calcium, phosphorus, magnesium, uric acid, creatinine amino acids Beta2-microglobulin, alpha1-microglobulin, or other low molecular weight proteins
Indicators calculated	glomerular filtration rate (GFR) Calcium-creatinine ratio in urine Phosphate-creatinine ratio in urine Fractional phosphate excretion in urine Tubular reabsorption of phosphate (TRP) Tubular maximal phosphate reabsorption related to glomerular filtration rate (TmP/GFR)

**Table 6:** Age and sex-dependent normal values for serum phosphate according to various sources (modified) [26,27].

Age (Years)	Boys	Girls
Nelson Textbook of Pediatrics, 22nd Edition [28]		
Neonates	1.55–2.65	1.55–2.65
Infants and toddlers < 3 years	1.25–2.10	1.25–2.10
Children 4–11 years	1.20–1.80	1.20–1.80
Adolescents >12 years	0.90–1.80	0.90–1.80
The HARP cohort [26]		
0 years	1.58–2.24	1.65–2.25
1 year	1.46–2.08	1.52–2.10
3 years	1.29–1.87	1.34–1.88
7 years	1.25–1.89	1.24–1.81
10 years	1.22–1.90	1.17–1.78
12 years	1.19–1.90	1.10–1.72
15 years	1.04–1.75	0.92–1.51
18 years	0.73–1.39	0.79–1.36
The CALIPER cohort [27]		
0–14 days	1.80–3.40	1.80–3.40
15 days–<1 year	1.54–2.72	1.54–2.72
1 year–<5 years	1.38–2.19	1.38–2.19
5 years–<13 years	1.33–1.92	1.33–1.92
13 years–<16 years	1.14–1.99	1.02–1.79
16 years–<19 years	0.95–1.62	0.95–1.62

**Table 7:** Causes of hypophosphatemia according to [25,30,32], modified.

Extrarenal Causes (Low Urinary Phosphate Loss)		Renal Causes (High Urinary Phosphate Loss)		
Intestinal Losses or Other Losses	Intracellular Shift	Intrinsic Renal Loss	PTH-Dependent Renal Loss	FGF23-Dependent Renal Loss
Vitamin D deficiency	Insulin	Congenital Fanconi syndrome	Primary hyperparathyroidism	X-linked hypophosphatemia
Diarrhea	Refeeding syndrome	Acquired Fanconi syndrome	Vitamin-D-deficient rickets	Autosomal dominant hypophosphatemic rickets
Malnutrition	Alkalosis	Pathogenic variants in *SCL34A1*, *SCL34A3*, and *CLCN5*	Vitamin-D-dependent rickets	Autosomal recessive hypophosphatemic rickets 1–3
Phosphate binders, antacids	Hungry bone syndrome		Jansen metaphyseal chondrodysplasia	Tumor-induced osteomalacia
				Ferric carboxymaltose infusion
				Autoantibodies against PHEX protein

**Table 8:** Calcium-phosphorus metabolism in the case of non-renal (e.g., gastrointestinal) phosphate losses according to [30,33,34].

Ca	P	ALP	PTH	25OHD	1,25(OH)_2_D	iFGF23	U_Ca_	U_P_	TmP/GFR
N/↑	↓	↑↑↑	N	N	N/↑	N	Variable	↓	N

Ca—serum calcium, P—serum phosphate, ALP—alkaline phosphatase, PTH—parathyroid hormone, 25OHD—25-hydroxyvitamin D, 1,25(OH)_2_D—1,25-dihydroxyvitamin (calcitriol), iFGF23—intact fibroblast growth factor 23, U_Ca_—urinary calcium, U_P_—urinary phosphate, TmP/GFR—tubular maximal phosphate reabsorption related to glomerular filtration rate, N—normal, ↑↑↑—very significantly elevated, ↓—lowered, ↑—elevated.

**Table 9:** Calcium-phosphorus metabolism in primary hyperparathyroidism according to [30,33,34].

Ca	P	ALP	PTH	25OHD	1,25(OH)_2_D	iFGF23	U_Ca_	U_P_	TmP/GFR
**↑**	**↓**	**↑**	**↑**	**N**	**N/** **↑**	**N**	**↑**	**↑**	**↓**

Ca—serum calcium, P—serum phosphate, ALP—alkaline phosphatase, PTH—parathyroid hormone, 25OHD—25-hydroxyvitamin D, 1,25(OH)_2_D—1,25-dihydroxyvitamin (calcitriol), iFGF23—intact fibroblast growth factor 23, U_Ca_—urinary calcium, U_P_—urinary phosphate, TmP/GFR—tubular maximal phosphate reabsorption related to glomerular filtration rate, N—normal, **↓**—lowered, **↑**—elevated.

**Table 10:** Calcium-phosphorus metabolism in calcipenic rickets according to [30,33,34].

Disease	Ca	P	ALP	PTH	25OHD	iFGF23	1,25(OH)_2_D	U_Ca_	U_P_	TmP/GFR
Vit. D deficiency	N/↓	N/↓	↑↑↑	↑↑↑	↓↓↓	N	Variable	↓	Variable	↓
VDDR1A	↓	N/↓	↑↑↑	↑↑↑	N	N/↓	↓	↓	Variable	↓
VDDR1B	↓	N/↓	↑↑↑	↑↑↑	↓↓	N	Variable	↓	Variable	↓
VDDR2A	↓	N/↓	↑↑↑	↑↑↑	N	N/↓	↑↑	↓	Variable	↓
VDDR2B	↓	N/↓	↑↑↑	↑↑↑	N	N	↑↑	↓	Variable	↓
VDDR3	↓	↓	↑↑↑	↑↑↑	↓	N	↓	↓	Variable	↓

Ca—serum calcium, P—serum phosphate, ALP—alkaline phosphatase, PTH—parathyroid hormone, 25OHD—25-hydroxyvitamin D, 1,25(OH)_2_D—1,25-dihydroxyvitamin (calcitriol), iFGF23—intact fibroblast growth factor 23, U_Ca_—urinary calcium, U_P_—urinary phosphate, TmP/GFR—tubular maximal phosphate reabsorption related to glomerular filtration rate, N—normal, ↑↑↑—very significantly elevated, ↓↓↓—very significantly lowered, ↑↑—significantly elevated, ↓↓—significantly lowered, ↑—elevated, ↓—lowered, VDDR—vitamin-D-dependent rickets.

**Table 11:** Vitamin D-dependent rickets according to [25,30,33,34].

Disease and OMIM Number	Gene	Inheritance	Estimated Prevalence According to OMIM and ORPHANET	Mechanism
VDDR1A, #264700	*CYP27B1*	AR	1–5/10,000	Disturbed 1,25(OH)_2_D synthesis
VDDR1B, #600081	*CYP2R1*	AR	1–5/10,000	Disturbed 25(OH)D synthesis
VDDR2A, #277440	*VDR*	AR	Unknown	Disturbed signal transduction from VDR
VDDR2B, #600785	*HNRNPC*	AR	Unknown	Disturbed signal transduction from VDR
VDDR3, #619073	*CYP3A4*	AD	Unknown	Excessive breakdown of 1,25(OH)_2_D

**Table 12:** Primary renal hypophosphatemia—PTH-independent and FGF23-independent causes according to [25,30,33,34].

Disease and OMIM Number	The Gene Responsible	Estimated Prevalence According to OMIM and ORPHANET	Characteristic Features
Infantile hypercalcemia-2 (HCINF2; #616963) Fanconi renal-tubular syndrome 2 (FRTS; #613388) Hypophosphatemic nephrolithiasis/osteoporosis-1 (NPHLOP1; 612286)	*SLC34A1*	<1/1,000,000	Loss of sodium-phosphate cotransporter NaPi2a function in the proximal tubule, decreased FGF23 concentration, high 1,25(OH)_2_D concentration
Hereditary hypophosphatemic rickets with hypercalciuria (HHHR); #241530	*SLC34A3*	1/250,000	Loss of sodium-phosphate cotransporter NaPi2c function in the proximal tubule, decreased FGF23 concentration, high 1,25(OH)_2_D concentration
Proteinuria, low molecular weight, with hypercalciuric nephrocalcinosis (#308990)	*CLCN5*	1/400,000–1,000,000	Loss of *CLCN5* function in the proximal tubule
Genetically determined Fanconi syndrome (e.g., cystinosis—#219800, #219900)	*CTNS* and others	1–9/100,000	Extrarenal symptoms may occur, such as the accumulation of cystine in the eye.
Acquired Fanconi syndrome	-	Unknown	Heavy metal poisoning, a condition after treatment with, among others, ifosfamide, cisplatin, and aminoglycoside antibiotics

**Table 13:** Metabolic disturbances in patients with pathogenic variants in *SLC34A1* or *SLC34A3* according to [30,33,34].

Ca	P	ALP	PTH	25OHD	1,25(OH)_2_D	iFGF23	U_Ca_	U_P_	TmP/GFR
**N**	**↓/N**	**↑**	**N/↑**	**N**	**↑/N**	**N/** **↓**	**↑/N**	**↑**	**↓**

Ca—serum calcium, P—serum phosphate, ALP—alkaline phosphatase, PTH—parathyroid hormone, 25OHD—25-hydroxyvitamin D, 1,25(OH)_2_D—1,25-dihydroxyvitamin (calcitriol), iFGF23—intact fibroblast growth factor 23, U_Ca_—urinary calcium, U_P_—urinary phosphate, TmP/GFR—tubular maximal phosphate reabsorption related to glomerular filtration rate, N—normal, **↓**—lowered, **↑**—elevated.

**Table 14:** Causes of Fanconi syndrome according to [50,51,52,53,54].

Inherited causes	Cystinosis Dent’s disease Fanconi–Bickel syndrome Galactosemia Glycogenosis type I (von Gierke disease) Hereditary fructose intolerance Mitochondrial cytopathies Lowe’s syndrome Lysinuric protein intolerance Tyrosinemia type I Wilson’s disease Idiopathic Fanconi syndrome
Acquired causes	Acute tubular necrosis Acute/chronic tubulointerstitial nephritis Sjögren syndrome Medications (aminoglycosides, tetracyclines, tenofovir, adefovir, cidofovir, didanosine, lamivudine, stavudine, ifosfamide, cisplatin, valproate, salicylates) Iron-chelating agents (deferasirox) Heavy-metal exposure (cadmium, lead, mercury, chromium) Monoclonal gammapathies Renal transplantation

**Table 15:** Metabolic disturbances in Fanconi syndrome according to [30,33,34].

Ca	P	ALP	PTH	25OHD	1,25(OH)_2_D	iFGF23	U_Ca_	U_P_	TmP/GFR
**N/** **↓**	**↓**	**↑**	**N/↑**	**N**	**N/** **↓**	**N/** **↓**	**↑**	**↑**	**↓**

Ca—serum calcium, P—serum phosphate, ALP—alkaline phosphatase, PTH—parathyroid hormone, 25OHD—25-hydroxyvitamin D, 1,25(OH)_2_D—1,25-dihydroxyvitamin (calcitriol), iFGF23—intact fibroblast growth factor 23, U_Ca_—urinary calcium, U_P_—urinary phosphate, TmP/GFR—tubular maximal phosphate reabsorption related to glomerular filtration rate, N—normal, **↓**—lowered, **↑**—elevated.

**Table 16:** Hypophosphatemia secondary to excess FGF23 according to [25,30,33,34].

The Disease OMIM Number	The Gene Responsible	Estimated Prevalence According to OMIM and ORPHANET	Characteristic Features
X-linked hypophosphatemic rickets (XLH) #307800	*PHEX*	1–9/100,000	Beginning in childhood, periapical lesions, rickets, enthesopathies, hearing loss, and often hypocalciuria
Autosomal dominant hypophosphatemic rickets (ADHR) #193100	*FGF23*	<1/1,000,000	Onset at various ages may be accompanied by periods of exacerbation and remission.
Autosomal recessive hypophosphatemic rickets, autosomal recessive hypophosphatemia ARHR1, #241520; ARHR2, #613312;	*DMP1, ENPP1*	1/64,000–1/250,000	ARHR1—phenotype similar to HXL, ENPP1—may manifest as generalized arterial calcification in infancy
Raine syndrome #259775	*FAM20C*	<1/1,000,000	Often lethal in the perinatal period, osteosclerosis
Tumor-induced osteomalacia (TIO)	In 60% of cases, the *FN1-FGFR1* or *FN1-FGF1* fusion gene	0.1–0.7/100,000	Different ages of onset, bone pain, pseudo-fractures, weakness, and tumors may be small and difficult to locate
McCune-Albright syndrome #174800	*GNAS1*	1/100,000–1/1,000,000	Aminoaciduria, proteinuria, fibrous dysplasia, café au lait spots, premature puberty
Cutaneous skeletal hypophosphatemia syndrome (CSHS)	*RAS* somatic mutation	Unknown, less than 100 cases reported	Skin marks, neurological defects
intravenous iron supply	n/a	n/a	Intravenous iron induces an increase in FGF23, pseudofractures
Autoantibodies against PHEX protein	n/a	5 cases reported	Described in Japanese adults, phenotype similar to TIO, other autoimmune diseases may be present

**Table 17:** Calcium-phosphorus metabolism in X-linked hypophosphatemia according to [30,33,34].

Ca	P	ALP	PTH	25OHD	1,25(OH)_2_D	iFGF23	U_Ca_	U_P_	TmP/GFR
**N**	**↓**	**↑**	**N/↑**	**N**	**N/** **↓**	**↑/** **N**	**↓**	**↑**	**↓**

Ca—serum calcium, P—serum phosphate, ALP—alkaline phosphatase, PTH—parathyroid hormone, 25OHD—25-hydroxyvitamin D, 1,25(OH)_2_D—1,25-dihydroxyvitamin (calcitriol), iFGF23—intact fibroblast growth factor 23, U_Ca_—urinary calcium, U_P_—urinary phosphate, TmP/GFR—tubular maximal phosphate reabsorption related to glomerular filtration rate, N—normal, **↓**—lowered, **↑**—elevated.

**Table 18:** Examples of causal treatment of hypophosphatemia in children.

Disease	Treatment Modality
Malabsorption due to celiac disease	Gluten-free diet
Malabsorption due to Giardiasis	Tinidazol, metronidazole
Malabsorption due to Whipple’s disease	Long-term antibiotic therapy—penicillin/ceftriaxone followed by trimethoprim/sulphamethoxazole
Hypophosphatemia due to removal in renal replacement therapy	Modification of dialysis protocol
Fanconi syndrome due to cystinosis	Cysteamine
Fanconi syndrome due to Sjögren disease	Immunosuppressive treatment
Parathyroid hormone-producing adenoma	Parathyroidectomy
Tumor-induced osteomalacia	Surgical treatment/burosumab
X-linked hypophosphatemia	Burosumab

## Data Availability

No new data were created.

## Supplementary Materials

The following supporting information can be downloaded at: https://www.mdpi.com/article/10.3390/biom15091321/s1, Supplementary Table S1: Calcium-phosphorus metabolism in different causes of hypophosphatemia according to [25,30,33,34].
