# Supplementary material for: Approach to a Child with Hypophosphatemia
Source: Biomolecules. 2025 Sep 15;15(9):1321. doi: 10.3390/biom15091321 (PMC12467876; doi:10.3390/biom15091321)
Supplement: Supplementary file 1 [file biomolecules-15-01321-s001.zip › biomolecules-3753147-supplementary.pdf]

Supplementary Table S1. Calcium-phosphorus metabolism in different causes of hypophosphatemia according to [25, 30, 33, 34].

Cause of hypo-phospha-temia	Ca	P	ALP	PTH	25OHD	1,25(OH)₂D	iFGF23	U_{Ca}	U_P	TmP/GFR
**Non-renal causes**	N/↑	↓	↑↑↑	N	N	N/↑	N	Variable	↓	N
**Primary hyper-parathy-roidism**	↑	↓	↑	↑	**N**	**N/↑**	**N**	↑	↑	↓
**Vit. D deficiency**	N/↓	N/↓	↑↑↑	↑↑↑	↓↓↓	N	Variable	↓	Variable	↓
**VDDR1A**	↓	N/↓	↑↑↑	↑↑↑	N	N/↓	↓	↓	Variable	↓
**VDDR1B**	↓	N/↓	↑↑↑	↑↑↑	↓↓	N	Variable	↓	Variable	↓
**VDDR2A**	↓	N/↓	↑↑↑	↑↑↑	N	N/↓	↑↑	↓	Variable	↓
**VDDR2B**	↓	N/↓	↑↑↑	↑↑↑	N	N	↑↑	↓	Variable	↓
**VDDR3**	↓	↓	↑↑↑	↑↑↑	↓	N	↓	↓	Variable	↓
**SLC34A1 or SLC34A3 patho-genic variants**	**N**	**↓/N**	↑	**N/↑**	**N**	**↑/N**	**N/↓**	**↑/N**	↑	↓
**Fanconi syndrome**	**N / ↓**	↓	↑	**N/↑**	**N**	**N/↓**	**N/↓**	↑	↑	↓
**X-linked hypo-phospha-temia**	**N**	↓	↑	**N/↑**	**N**	**N/↓**	**↑/N**	↓	↑	↓

Ca – serum calcium, P – serum phosphate, ALP – alkaline phosphatase, PTH – parathyroid hormone, 25OHD – 25-hydroxyvitamin D, $1,25(OH)_2D$ – 1,25-dihydroxyvitamin (calcitriol), iFGF23 – intact fibroblast growth factor 23, U_{Ca} – urinary calcium, U_P – urinary phosphate, TmP/GFR – tubular maximal phosphate reabsorption related to glomerular filtration rate, N – normal, ↓ – lowered, ↑ – elevated, VDDR – vitamin-D-dependent rickets
